# Autoclaved Tumor Bone for Skeletal Reconstruction in Paediatric Patients: A Low Cost Alternative in Developing Countries

**DOI:** 10.1155/2013/698461

**Published:** 2013-12-18

**Authors:** Masood Umer, Hafiz Muhammad Umer, Irfan Qadir, Haroon Rashid, Rabia Awan, Raza Askari, Shamvil Ashraf

**Affiliations:** Department of Orthopaedic Surgery, Aga Khan University Hospital, Stadium Road, Karachi 74800, Pakistan

## Abstract

We reviewed in this series forty patients of pediatric age who underwent resection for malignant tumors of musculoskeletal system followed by biological reconstruction. Our surgical procedure for reconstruction included (1) wide en bloc resection of the tumor; (2) curettage of tumor from the resected bone; (3) autoclaving for 8 minutes (4) bone grafting from the fibula (both vascularized and nonvascularized fibular grafts used); (5) reimplantation of the autoclaved bone into the host bone defect and fixation with plates. Functional evaluation was done using MSTS scoring system. At final followup of at least 18 months (mean 29.2 months), 31 patients had recovered without any complications. Thirty-eight patients successfully achieved a solid bony union between the graft and recipient bone. Three patients had surgical site infection. They were managed with wound debridement and flap coverage of the defect. Local recurrence and nonunion occurred in two patients each. One patient underwent disarticulation at hip due to extensive local disease and one died of metastasis. For patients with non-union, revision procedure with bone graft and compression plates was successfully used. The use of autoclaved tumor grafts provides a limb salvage option that is inexpensive and independent of external resources and is a viable option for musculoskeletal tumor management in developing countries.

## 1. Introduction

Skeletal reconstruction for large segmental defects following bone tumour extirpation has always been considered as a therapeutic challenge [1]. Most bone tumor patients are young and active; any given treatment must not only preserve the affected limb but also maintain function without major complications or reoperations over the long term [2]. After the resection of malignant tumors in children, a variety of reconstructive procedures have been used on a case-by-case basis, including rotationplasty [3, 4], arthrodesis, bonelengthening [5], extendable prostheses [6, 7], allografting [8], extracorporeal irradiated autografts [9, 10], vascularized or nonvascularized grafts [11], pasteurization [12], autoclaved bone [13], and amputations [14].

Prosthetic reconstruction allows rapid restoration of mobility and weightbearing. Its long-term results in pediatric patients are more controversial: infection and aseptic loosening occur commonly and require further surgery [15]. Biological reconstruction with an allograft or an autograft restores bone stock and allows better soft-tissue attachment compared with metal implants [16]. However, complications like risk of fracture (7% to 42%), nonunion (9% to 63%), and infection (5% to 30%) can be disturbingly high [17–19]. Moreover, allograft reconstruction depends on the availability of a bone bank so that the size of the allograft can be matched to that of the resected bone segment. In many Asian countries, bone allografts are often avoided for religious, social, and cultural reasons.

From a developing nation's perspective, reimplantation of extracorporeally devitalized tumor bearing bone segments is an appealing option. It allows immediate and anatomical correct filling of the defect. It is technically and financially a simple, cost-effective, and viable solution for these difficult problems [20]. This procedure consists of wide en bloc resection of the tumour, curettage of tumour tissue from the resected bone, extracorporeal bolus single irradiation or autoclaving, followed by reimplantation into the recipient with a fixation device. Serious problems remain, however, including nonunion, infection, and fracture. Combined use of autoclaved bone and vascularised bone would seem to be the ideal graft for reconstruction because of the cumulative advantages arising from this approach.

From a pediatric perspective, all the above methods of reconstruction are associated with problems such as deformation, growth retardation, limb length discrepancy, measures to be taken to cope with high levels of physical activity in childhood, and problems related to social adaptation. In the present study, we reviewed forty pediatric patients from our institute who underwent resection for malignant musculoskeletal tumour and were then reconstructed using a combination of vascularised bone graft and autoclaved autograft. We report here the clinical outcomes including radiographic findings, functional analyses, and the complications arising, as well as a discussion of the advantages and disadvantages of this approach.

## 2. Patients and Methods

We retrospectively reviewed 40 consecutive pediatric patients, aged 16 years or younger, with locally aggressive or malignant bone tumors treated with tumor resection, autoclaving, and reimplantation of the orthotopic autograft. Patients with intra-articular extension of tumor were excluded. All patients came from pediatric population and underwent surgical management at the Aga Khan University Hospital from January 2008 through June 2012.

The demographic data and clinical characteristics of the study population were acquired from clinical chart review, tumor registry information, physicians' records, patients' correspondence, and telephone interviews.

Our population included 23 males and 17 females. All patients were of pediatric age group with age ranging from 6 to 16 years. Osteosarcoma (57.5%) was the commonest followed by Ewing's sarcoma (37.5%). There were 2 cases of chondrosarcoma. Long bones were mainly involved with most common involvement of lower limb. There were 17 cases of femur and 14 of tibia. From upper limb, there were 5 cases of humerus and 3 of radius. Only one case involved calcaneum of left side. Mean resection length was 13.9 cm (range: 0–28.1 cm) and mean length of reimplanted autoclaved graft was 18.5 cm (range: 0–32.3 cm).

Full oncological staging of each patient was performed before planning surgery, including biopsy. Multidisciplinary protocol was used to determine timing of tumor resection. For those patients who had osteosarcoma and Ewing's sarcoma, adjuvant and neoadjuvant chemotherapy were administered according to international protocols [21].

### 2.1. Surgical Procedure

An adequate margin of osteotomy was determined by radiography using magnetic resonance imaging. In case of metaphyseal tumors, osteotomy was performed just proximal/distal to the physis and also saving the intra-articular part of the involved bone thereby preserving the joint. The method used by the authors consists of (1) wide en bloc resection of the tumour; (2) curettage of tumour from the resected bone; (3) autoclaving at 130° for 8 minutes (4) bone grafting from the fibula (both vascularized and nonvascularized fibular grafts used); (5) reimplantation of the autoclaved bone into the host bone defect and fixation with plates and/or IM nail [22]. To ensure an adequate surgical margin, frozen sections of multiple relevant and representative biopsy specimens were obtained.

The specimens were heated in an autoclave machine at 130° for 8 minutes [22]. Upon removal from the autoclave, the remaining soft tissue was easily scraped off from the surface of the bone. The specimen was then soaked in antibiotic mixed normal saline for 5 minutes and prepared for reinsertion. The whole process was performed under sterile conditions, with sterile wraps used for transport between the surgical field and the autoclave machine. This autoclaved segment of bone was then supplemented with either a vascularised or nonvascularised fibular bone graft and fixed to the host bone with metal plates and screws. The fibular graft length was always 3-4 cm more than the resection length. This larger fibular strut was used for a two-centimeter overlap at both proximal and distal ends of involved bone.

### 2.2. Clinical Evaluation

After operation those patients who had undergone a femoral reconstruction used crutches with partial weight bearing initially until the graft had united and the limb was considered to be stable. In tibial reconstruction, where the patellar tendon had been reattached, the lower limb was immobilised in a cast, usually for six weeks. Only passive movements were allowed for six weeks to be followed by active range of motion at the knee. When radiological healing was present at the site of the osteotomy, unassisted walking with full weight bearing was allowed. Limb length assessment was done using standing scanogram (lower limb standing long film). In the humeral reconstruction, the upper limb was supported by a sling postoperatively. Passive movements of the shoulder and elbow were allowed after two weeks. Objects more than 2 kg were not lifted until there was radiological union at the osteotomy site.

The patients were followed up every six weeks to evaluate the healing of the osteotomy, functional recovery, and potential complications until union and then every three months thereafter. The site of the osteotomy was considered to be healed radiologically if callus was seen bridging the site in both the anteroposterior and lateral planes. Nonunion was defined as failure of union one year after operation. Functional evaluation was assessed using the Musculoskeletal Tumour Society (MSTS) scoring system [23] which includes pain, function, patient acceptance, need for external supports, walking ability, and gait.

## 3. Results

**Figure 1:**
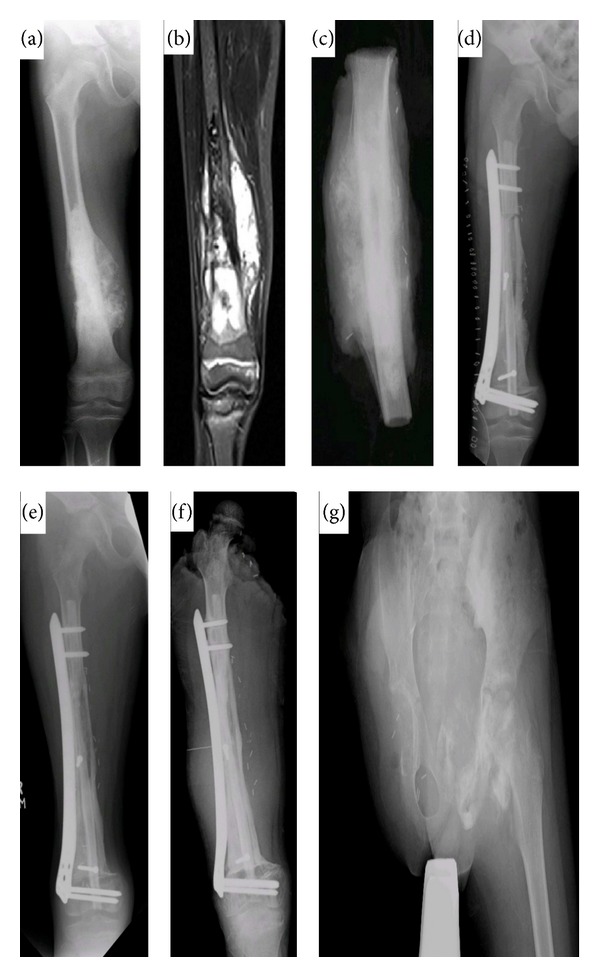
A 10-year-old boy with osteogenic sarcoma. (a) X-ray shows lytic mass in right distal femur. (b) MRI shows osteoid lesion in the metadiaphyseal region of right distal femur. (c) X-ray of the resected bone (d) Immediate postoperative X-ray. (e) X-ray shows graft and host union at both proximal and distal ends 10 months post-operatively. (f) 3-year postoperative X-ray shows local recurrence. (g) X-ray after disarticulation of right hip.

At the final followup of at least 18 months (mean 29.2 months), 31 patients had recovered without any complications. Thirty-eight patients successfully achieved a solid bony union between the graft and recipient bone. Bony union was initially achieved between recipient bone and vascularized bone graft, followed by vascularized bone graft and autoclaved bone, and finally between recipient and autoclaved bone. One patient died due to distant metastasis to pelvis and lungs. Overall, mean time to full weight bearing was 6 months whereas mean time to complete bony union was 9.5 months. Mean MSTS score was 22. Details on individual cases can be found in Table 1 and Figure 1.

### 3.1. Early Complications

Three patients had deep infection at surgical sites. They were treated with systemic antibiotics along with surgical debridement. Two of these patients also required a coverage procedure with a gastrocnemius flap. One patient had significant skin necrosis at the surgical site. The gastrocnemius flap also failed and a free latissimus dorsi flap was used successfully to cover the defect.

### 3.2. Late Complications

**Figure 2:**
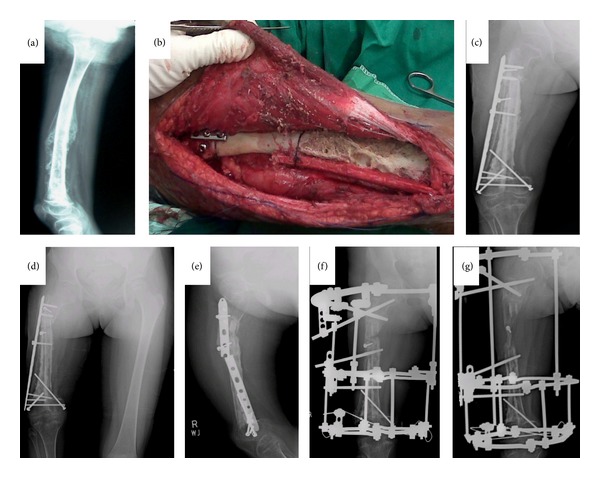
A 10-year-old girl with Ewing's sarcoma. (a) X-ray shows mass in mid and distal right femur. (b) Intraoperative picture showing autoclaved bone with fibular graft. (c) Immediate postoperative X-ray (d) 26-month postoperative X-ray shows graft and bone union at both distal and proximal ends; however, there is considerable shortening of right femur. (e) Breakage of locking compression plate (f) Considering the limb length discrepancy and plate breakage, patient underwent Ilizarov application to right femur. (g) One year post-Ilizarov X-ray showing 2.5 cm gain in right femur length.

Two patients experienced pathological fractures at surgical site at the 23rd and 29th months. Both underwent intramedullary nailing for fracture fixation. Two patients experienced local recurrence at 25th and 32nd months. One patient had to undergo disarticulation at hip due to extensive involvement of adjacent neurovascular structures and soft tissue. An other patient underwent wide margin reexcision. Two patients had nonunion at osteotomy sites. Revision procedures were done in both cases after waiting for one year. The procedure included bone graft usage and compression plating for fixation (Figure 2).

## 4. Discussion

When deciding which reconstructive procedure is the best after resection of a tumour, the surgeon must consider the applicability of the procedure, the level of difficulty, the patient's age and functional demands, and the morbidity and incidence of complications as well as durability of the reconstructive procedure [16].

From a developing nation's perspective, where patients themselves are primary payers of medical services, the debate still moves around the most cost-effective method of treatment. The technique of excision, sterilization, and reimplantation has the advantage of being a biological reconstruction with the potential for long-term survival. It is cheap and convenient and requires only one surgeon, and the operating time is much shorter than other reconstructive procedures. In the present study, by autoclaving and reusing the patient's own bone, we obviated the need to procure an allograft which poses substantial problems in a country like Pakistan. In Pakistan, bone banking facilities are not available and are unlikely to be established in the near future because of financial, religious, and cultural constraints.

Primary advantages of autoclaved reimplants are that the implanted bone segment is a “custom fit” being the patient's, own bone; no bone banking techniques, costs, immunological response, or transmission risks are involved; and they provide anatomical reattachment of muscles and tendons and natural joint preservation, with a higher incidence of incorporation and peripheral healing than allografts [24]. Definite removal of all tumor tissue is the most important factor in the surgical treatment of bone tumors with curative intention. Therefore, conscientiously performed biopsies to ensure an adequate surgical margin are of crucial importance [25].

The only real requirement is that the bone that is excised should have sufficient structural strength to be reinserted after sterilization, when of course it will “fit perfectly” [26, 27]. On the other hand, reimplanting an autoclaved bone is like using cadaveric bone, which presumably has no biological activity. For our pediatric patients we reduced the autoclaving time to 8 minutes. Autoclaving tumor bones for 10 minutes, as we do for adults [22], was making their bones too soft and unable to provide any structural strength. Our recurrence rate has not been affected by reducing this autoclaving time. Over time, resorption will often result in a stress fracture or mechanical failure of the construct. The purpose of the vascularised fibular graft is to improve the blood supply to the osteotomy sites and thereby minimise the time to union, reduce bone resorption, and improve structural stability [28].

Other techniques that are capable of destroying tumour cells in resected bone include single dose bolus radiotherapy, pasteurization, and repeated freezing-thawing with liquid nitrogen. Singh et al. [29] conducted a study to evaluate the effect of several sterilization methods, including autoclaving, boiling, pasteurization, and irradiation, on the mechanical behavior of human cortical bone graft and histopathology evaluation of tumour bone samples. They concluded that all methods of sterilization gave rise to 100 percent tumour kill; however, main difference laies in their effect on mechanical properties of bone.

An alternative for reconstruction following skeletal tumour resection is allografting. Chen et al. [16] compared the clinical outcome between groups with segmental allografts and devitalized (irradiated) autografts. No significant difference between the groups was found; however, the complication rate was unexpectedly high. Nonunion and late fracture occurred in 7% and 20%, respectively, of the irradiated bone group and in 43% and 14%, respectively, of the allograft group. In an attempt to reduce these complications, the present study used fibular bone grafts with autoclaved bone. Due to technical reasons, we could not use vascularized fibula in all the patients. However, non-vascularized fibula also worked almost equally nicely and was incorporated quickly. Supplementing with a fibula led to early union and is recommended as an important adjunct to autoclaved bone.

To our knowledge, only a few studies have attempted to enhance the neovascularisation of necrotic autoclaved autogenic or allogeneic bone grafts by combining these with vascularized bone grafting. Chang and Weber [30] used vascularised fibula graft and allograft in 14 cases to reconstruct massive diaphyseal bone defects following tumour resection. In their study, all but one case achieved successful bony union without late fatigue fracture of the fibula graft. These workers concluded that combined use could prevent allograft nonunion and result in decreased time to bony union. Sunagawa et al. [31] have previously demonstrated a neovascularization effect of vascularised bone graft to necrotic bone autograft in a canine model. The rationale for a combined vascularised and devitalized bone autograft is the cumulative advantage provided by mechanical endurance from the latter with the biological properties of the former [32].

## 5. Conclusion

The management of primary malignant bone tumors in less developed countries is often a daunting task, strewn with a long list of complications. This study is limited by the small sample size and short followup; however, we conclude that use of autoclaved tumor grafts provides a limb salvage option that is inexpensive and independent of external resources without sacrificing appropriate oncologic principles. Longer followups are needed to assess the long-term complications such as limb length discrepancy and its subsequent management.

## Figures and Tables

**Table 1:** Baseline characteristics of patients.

Sr./sex/age (years)	Side/site	Diagnosis	Resection length	Length of autograft	Method of fixation	Length of surgery (min)	Length of hospital stay	MSTS	Time to FWB (months)	Time to bone union (months)	Complication	Second procedure
1/F/8	R/femur	Osteosarcoma	28.1	32.2	LCP	150	7	26	6	8	—	—
2/F/15	R/humerus	Ewing's sarcoma	20.1	21.0	LCP	120	5	19	—	—	Nonunion	Graft and LCP
3/M/6	L/tibia	Ewing's sarcoma	12.3	18.2	LCP	260	10	20	7	10	—	—
4/F/13	R/femur	Osteosarcoma	16.7	25.0	LCP	245	7	20	8	10	—	—
5/M/12	R/femur	Osteogenic sarcoma	17.2	24.6	LCP	240	8	28	6	9	Local recurrence	Hip disarticulation
6/M/10	R/distal Radius	Osteosarcoma	7.9	9.6	K-wire	177	5	27	—	8	—	—
7/M/15	R/femur	Ewing's sarcoma	9.6	14.3	LCP	260	7	21	3	5	—	—
8/M/15	L/tibia	Ewing's sarcoma	17.1	18.0	LCP	310	6	19	4	11	—	—
9/F/7	L/tibia	Chondrosarcoma	15.2	19.1	LCP	300	7	20	5	6	—	—
10/F/13	R/femur	Osteogenic sarcoma	16.3	23.3	LCP	447	10	22	13	36	Surgical site infection	Wound Debridement and gastroecnemius flap
11/M/6	L/humerus	Ewing's sarcoma	8.8	14.1	Screws and X-fix	270	6	26	—	8	Distant metastasis	—
12/F/14	L/tibia	Osteogenic sarcoma	8.2	16.7	LCP	240	7	22	4	11	—	—
13/M/11	L/humerus	Osteosarcoma	6.4	7.1	LCP	230	6	23	—	5	—	—
14/M/11	R/tibia	Osteogenic sarcoma	15.3	23.1	X-fix	190	5	20	6	11	Surgical site infection	Gastroecnemius flap
15/F/7	R/femur	Osteosarcoma	21.3	25.8	LCP	345	7	20	4	8	—	—
16/F/8	L/tibia	Osteogenic sarcoma	18.1	21.5	LCP	390	5	26	5	9	—	—
17/F/12	L/tibia	Osteogenic sarcoma	15.4	22.5	LCP	395	8	21	3	5	—	—
18/M/9	R/femur	Osteogenic sarcoma	16.7	26.5	LCP	135	6	26	8	13	—	—
19/F/12	R/femur	Ewing's sarcoma	16.2	19.6	LCP	210	7	21	7	—	Nonunion	Graft and LCP
20/M/10	R/femur	Osteogenic sarcoma	13.0	17.6	LCP	260	8	22	5	6	—	—
21/F/11	R/humerus	Ewing's sarcoma	13.2	14.9	LCP	185	7	22	—	6	—	—
22/M/13	R/femur	Osteogenic sarcoma	15.0	24.0	LCP	310	8	22	9	13	—	—
23 /M/13	L/tibia	Ewing's sarcoma	13.1	16.0	LCP	160	7	18	4	11	Local recurrence	Wide margin excision
24/M/14	R/femur	Ewing's sarcoma	20.2	24.3	LCP	280	8	22	5	6	—	—
25/M/8	R/radius	Ewing's sarcoma	5.7	7.1	LCP	180	5	26	—	8	—	—
26/F/15	R/femur	Ewing's sarcoma	24.7	28.8	LCP	240	8	21	12	25	Skin necrosis	Free flap
27/M/16	L/tibia	Osteosarcoma	16.1	19.1	LCP	260	7	18	7	9	—	—
28/F/16	R/humers	Osteosarcoma	10.0	12.6	X-fix	177	8	20	—	9	—	—
29/F/16	L/calcaneum	Ewing's sarcoma	—	—	Screws	120	5	21	—	13	—	—
30/F/8	R/femur	Osteosarcoma	15.0	19.0	LCP	240	8	16	8	12	Fracture	I.M nail
31/M/16	L/femur	Osteogenic sarcoma	6.6	9.7	Screws	200	7	20	5	6	—	—
32/M/16	L/tibia	Osteogenic sarcoma	20.6	24.1	LCP	240	8	22	6	9	—	—
33/M/7	L/tibia	Chondrosarcoma	16.4	21.1	LCP	245	7	25	4	9	—	—
34/M/15	R/tibia	Osteosarcoma	10.9	16.3	LCP	219	6	21	5	10	—	—
35/M/10	L/femur	Ewing's sarcoma	18.0	21.0	LCP	255	8	23	8	14	—	—
36/M/7	R/radius	Ewing's sarcoma	8.7	10.0	LCP	240	7	21	—	7	—	—
37/F/12	R/femur	Osteogenic sarcoma	11.8	15.0	LCP	210	7	23	7	12	Fracture	I.M nail
38/M/11	R/tibia	Osteosarcoma	13.6	21.0	LCP	240	8	27	4	8	—	—
39/F/12	L/femur	Osteosarcoma	18.9	23.1	LCP	255	7	25	4	10	—	—
40/M/9	L/tibia	Ewing's sarcoma	11.4	15.0	LCP	230	8	22	7	11	—	—

M: male; F: female; L: left; R: right; LCP: locking compression plate; I.M nail: intramedullary nail.
